# Assessment of Primary Care Physicians’ Expertise of Common Dermatological Conditions in the Jouf Region, Saudi Arabia: A Mixed Methods Study

**DOI:** 10.3390/healthcare11121705

**Published:** 2023-06-10

**Authors:** Hatem M. Alotaibi, Ziyad M. Alruwaili, Ahmed A. Dilli, Abdullah A. Altaleb, Mohanad M. Asiri, Osama J. Alwadani, Ziad M. Alshaalan, Umar-Farooq Dar

**Affiliations:** 1 College of Medicine, Jouf University, Sakaka 72388, Saudi Arabia; 2 Division of Dermatology, Department of Medicine, College of Medicine, Jouf University, Sakaka 72388, Saudi Arabia; 3 Department of Family & Community Medicine, College of Medicine, Jouf University, Sakaka 72388, Saudi Arabia; ufdar@ju.edu.sa

**Keywords:** primary care physicians, dermatology, diagnosis, management, referrals

## Abstract

Primary care physicians (PCPs) are the first line of defense for the management of common dermatological conditions (DCs). This study aimed to assess how dermatological diseases are identified, managed, and referred to in primary healthcare centers (PHCs). This was a mixed methods study comprising a cross-sectional survey and semi-structured interviews recruited through PHCs across the Jouf region of Saudi Arabia. Sixty-one PCPs completed the data, and eight participants were interviewed. A survey based on a sample of 22 photographs of common DCs in the Kingdom was administered to the participants to answer questions about the correct diagnosis, appropriate management, referral strategy, and encounter rate. In our sampled population, the mean overall knowledge level on a scale of 10 was 7.08 (±1.3). Among participants that had good to acceptable scores, 51 (83.6%) were in the overall knowledge parameter, 46 (75.4%) in the diagnosis parameter, and 49 (80.3%) in the management parameter. PCPs with five years or more of experience were found to have significantly higher overall knowledge and management scores. Most of our PCPs demonstrated sufficient knowledge of common DCs and had good to acceptable scores in all parameters. However, educational and regulatory aspects of PCPs’ clinical management were identified. Focused training, provision of workshops, and improving medical school curricula regarding common DCs are recommended.

## 1. Introduction

Dermatological conditions (DCs) represent a significant burden worldwide. DC consultations account for approximately 25% of all primary care physicians (PCPs) consultations [1]. PCPs (i.e., general practitioners and family physicians) play an essential role in the management of common DCs in their region, while paying attention to specialist referral criteria and being familiar with commonly prescribed medications for managing such conditions [2].

DCs’ incidence and prevalence are primarily correlated with the ethnicity and genetic constitution of the community. For instance, a recent systematic review and meta-analysis explored the most common groups of DCs in Saudi Arabia: dermatitis and eczema, disorders of skin appendages, and skin infections, with pooled prevalence rates of 24%, 24.8%, and 18.5%, respectively [3]. Although common DCs are conceivably managed in a primary health care (PHC) setting, they account for almost 80% of dermatological consultations implemented by PCPs [4].

PCPs play a critical role in patient access to secondary and tertiary health care since they are the primary source of referrals to specialists [5]. Dermatologists comprise 8.2% of all medical specialists’ referrals, placing them among the medical specialists most commonly referred to by PCPs [1,5]. Prior to the dermatology clinic visit, most patients visit PCPs for the same complaints [6]. Furthermore, fewer services provided by PCPs for patients with DCs may suggest why most patients apply directly to secondary and tertiary health care centers for skin-related conditions [6].

PCPs are gatekeepers who are often the first to encounter patients with DCs [7]. Due to this vital responsibility, PCPs have been found to employ a set of diagnostic strategies, such as spot diagnosis, stepwise refinement [8], and pattern recognition for skin diseases, in order to carry out their role as per the American Academy of Family Physicians (AAFP) guidelines [9]. Since most common DCs can be easily recognized, complex diagnostic approaches are typically not required [5].

According to the AAFP, a PCP must know different diagnoses associated with different lesion types [9]. Despite this, PCPs have been found to refer patients to dermatologists with uncommon, as well as common, straightforward, skin conditions [10].

Despite the need for PCPs to keep updated on medical knowledge, it has been shown that more than two-thirds (68.5%) had insufficient knowledge of common DCs, and half were incompetent in managing skin disorders [11]. A lack of dermatological training is an essential factor responsible for difficulties in diagnosing and managing skin diseases. Moreover, appropriate training is believed to qualify PCPs to more precisely diagnose and manage skin conditions [1,2,3,4,5,6,7,8,9,10,11,12,13]. Additionally, an important study performed in Saudi Arabia showed that PCPs with a short period of specific clinical training in dermatology perform better in identifying and managing skin disorders than those without training [13]

Dermatological diseases are one of the most common diseases to present to PCPs, yet this group of diseases is often overlooked in medical education and training. Our research aimed to assess how dermatological diseases are specifically identified, managed, encountered, and referred to in the PHC setting. Additionally, we investigated the barriers and facilitators in PHC settings facing PCPs.

However, evaluating the PCPs’ clinical management of specific DCs will contribute to identifying areas of weakness in managing such conditions. Therefore, this needs assessment may help formulate evidence-based training for PCPs in the region and provide the basis for further research on the capability of PHCs in Saudi Arabia in managing common DCs.

## 2. Materials and Methods

### 2.1. Design, Settings, and Participants

This was a mixed methods study comprising a cross-sectional survey and semi-structured interviews. An explanatory sequential mixed methods design was used to overcome some of the study limitations, drive a holistic inquiry of the research question, and reach a complementary view of the findings, which reflect real-life practices of PCPs.

The survey study was conducted in PHCs across the Jouf region, Saudi Arabia. PCPs working at PHCs were invited to participate in the study. All steps and methods were performed in accordance with the relevant guidelines and regulations.

### 2.2. Inclusion and Exclusion Criteria

All PCPs working at PHCs in the Jouf region who were willing to participate in the study and on duty during data collection were recruited. PCPs who declined the participation invitation and those not on duty during data collection were excluded.

The study was carried out over four months, from 1 November 2021 to 28 February 2022.

### 2.3. Sample Size

The survey’s sample size was calculated based on the level of sufficient knowledge of DCs among PCPs of 31.5% [11] with a 5% margin of error and 95% confidence interval; the estimated sample size was rounded off to 103 participants as the total population size was 137. Recruitment was performed using a consecutive non-probability sampling technique. Eighty-three PCPs were contacted, and sixty-one participants completed the survey, for a response rate of 73.5%.

For the qualitative interviews, a purposeful sample of PCPs (n = 4) from the largest PHC in the region and dermatologists (n = 4) from the two main secondary healthcare centers in the region was included.

### 2.4. Data Collection

#### 2.4.1. The Cross-Sectional Survey

Data were collected through a semi-structured self-administered questionnaire developed based on the review of the relevant literature and distributed via direct contact with participants. The selection of cases was based on a recent systematic review and meta-analysis of the most common dermatological conditions in Saudi Arabia [3].

Validation of the content was performed through standardized photos and prototype presentations for all cases using the open source DermNetNZ.org (accessed on 3 August 2021) [14]. The AAFP standards for diagnosis, management, and referral criteria were also used to assess participants’ responses, as they are proposed to represent the bases of core clinical dermatology [9]. Reliability and internal consistency were measured using Cronbach’s alpha, which had a value of 0.78. The questionnaire comprised the following sections:

#### Part I, Personal Information

1. *Sociodemographic aspects include* age, gender, nationality, last qualification, years in practice, and current position.

2. *Practice in Dermatology:* number of dermatological cases encountered by the physician each month, referral probability, interests in dermatology.

##### Part II, Management of Dermatological Cases Assessment Tool

The study instrument was a photo quiz containing 22 photographs of the most common dermatological conditions in Saudi Arabia [3]. Classification of diagnoses was executed according to the *ICD-10*, and categorization into groups and subgroups was also performed to simplify the study, as shown in Table 1.

A short appropriate vignette accompanied each of the 22 cases to assess physicians’ history expertise. Participants were asked to identify the lesion, choose appropriate management, make referral decisions, and indicate if they had previously encountered such a case.

###### Assessment of Participants’ Answers

Participants’ responses were scored and adjusted on a scale of 0 to 10. A score of ≥8 was considered to be good, 6 -< 8 to be acceptable, and <6 to be low. The level of knowledge is defined as the sum of mean correct diagnoses and clinical management plans’ percentages on the photo quiz. Evaluation of the overall level of knowledge of PCPs was calculated as the mean of total proportions of correct diagnoses and management plans for all cases.

#### 2.4.2. The Qualitative Interviews

Following the survey, participants were contacted to schedule appointments and meetings to conduct the interviews. The interviews were conducted in participants’ clinics (n = 6) or offices (n = 2). Based on the relevant literature, the authors developed two semi-structured interview guides (Box 1) for PCPs and dermatologists. Each interview lasted from 10 to 20 min. The authors conducted face-to-face interviews using semi-structured interview guides (Box 1). The audio of the interviews was recorded with participants’ permission and transcribed verbatim for thematic content analysis.

Box 1. **Interview topic guide.**

**For Primary Care Physicians**

*Sociodemographic data*

- How old are you?
- What is your last qualification?
- What is your current position?
- How many years of practice do you have?
- What are your clinical interests?
- Have you had extracurricular training in dermatology?

*Practice data*

- What resources did you rely on to confirm your diagnosis and management when you faced a dermatological condition?
- When would you refer a patient, and on what basis do you make your decision?
- Usually, what medications do you prescribe for a dermatological condition? And are they available or covered by MOH?
- How would you describe your confidence when dealing with dermatological conditions?
- What difficulties do you face regarding diagnosing, managing, and providing medical advice?

*Ways for improvement*

- What are the needed facilities and services at PHCs to provide the best care for patients with dermatological conditions?
- Do you think that PCPs are well qualified to deal with common dermatological conditions? How can we improve their proficiency with such conditions?

**For Dermatologists**

*Sociodemographic data*

- How old are you?
- What is your last qualification?
- What is your current position?
- How many years of practice do you have?

*Practice data*

- How diverse are the referred cases you receive? And do they need specialist intervention?
- What do you think are the reasons some patients go directly to a dermatologist without visiting their PCP?
- How do you evaluate the current process of patient transfer from primary to secondary specialist care in MOH facilities?
- Do you face any cases that have been improperly managed by a PCP that led to complications? If yes, how do we fix such misconduct?

*MOH: Ministry of Health. PHCs: Primary Healthcare Centers. PCPs: Primary Care Physicians.*

The authors identified and coded themes and categories from interview transcripts. A comprehensive coding manual was generated to provide systematic data coding and negative case analysis. Confirmation of the generated codes was undertaken through respondent validation by returning to study participants to review and validate the analyses.

### 2.5. Statistical Analysis

Data were analyzed using SPSS version 22.0. Continuous data are presented as means and standard deviations (SDs). Categorical data are presented as counts and percentages. An independent samples *t*-test and Spearman’s correlation test were used to compare the continuous data, and Pearson’s chi-square test was used to compare the categorical data. The statistical significance of various cases in the photo quiz between the adequate knowledge and poor knowledge groups was assessed by the Mann–Whitney U test. A *p*-value of 0.05 was considered statistically significant in this study.

## 3. Results

### 3.1. Respondents’ Characteristics

Out of 61 PCPs, 44.26% (n = 27) were general practitioners and 55.73% (n = 34) were family medicine specialists in the Jouf region who completed the survey. Of the 61 PCPs, 62.3% were males, and 59% had a formal postgraduate qualification, out of which 41.6% were board-certified family physicians. The mean years in practice was 12 (±7), with a mean number of DC consultations per month of 27 (±25). Finally, less than half (42.6%) of the PCPs had training in dermatology. Other characteristics are shown in Table 2.

Furthermore, the interviewees’ mean age was 37 (±9.3) for PCPs and 34 (±3.9) for dermatologists. The mean years of experience for PCPs was 10.25 (±7.9), and for dermatologists it was 7.5 (±3.7). All interviewees (n = 8) were males except for PCP4, who is a female family physician.

### 3.2. Knowledge Level

**Figure 1:**
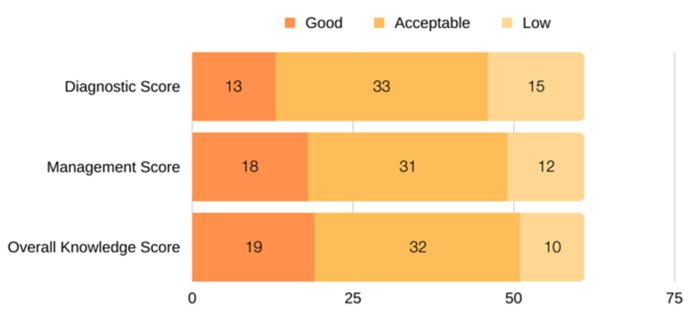
Distribution of participants throughout the overall knowledge, diagnosis, and management score categories. A score of ≥8 was considered good, 6 < 8 acceptable, and <6 low.

The mean overall knowledge level on a scale of 10 was 7.08 (±1.3). Among participants that had good to acceptable scores, 51 (83.6%) were in the overall knowledge parameter, 46 (75.4%) in the diagnosis parameter, and 49 (80.3%) in the management parameter (Figure 1). Additionally, Figure 1 demonstrates participants’ knowledge, diagnosis, and management categories, where a score of ≥8 was considered to be good, 6 -< 8 to be acceptable, and <6 to be low. However, regarding the knowledge level, approximately one-third (31.1%) of participants had good scores, more than half (52.5%) had acceptable scores, and only 16.4% had low scores.

Furthermore, PCPs with five years or more of experience were found to have significantly higher scores of overall knowledge and management (*p* = 0.042 and *p* = 0.032, respectively) (Table 3). However, there was no association of gender, postgraduate qualification, and training in dermatology with correct answers in overall knowledge, diagnosis, and management parameters.

In particular, the highest overall knowledge, of 87.3%, was about disorders of skin appendages, followed by papulosquamous disorders, dermatitis and eczema, and skin infections, with knowledge levels of 77.87%, 76.93%, and 71.5%, respectively. In addition, Table 4 demonstrates the groups and subgroups of the 22 DCs regarding proportions of disease recognition, management plans, referral decisions, encountering rates, and overall knowledge levels.

PCPs were asked about their opinion of the current status of PCPs’ expertise of DCs, and their responses ranged from not well qualified to well qualified. *“On common dermatological conditions, they might be somewhat qualified, but on the scale of all dermatological diseases, I do not think they are qualified enough”* (PCP 1, male senior registrar family physician, five years of experience).

### 3.3. Disease Recognition

PCPs’ provisional diagnoses of the photographed DCs varied throughout the assessed cases. However, the mean diagnosis score was 7.01 (±1.51), while the proportion of PCPs with correct diagnoses was 70.12%. Almost all PCPs (98.4%) correctly diagnosed acne vulgaris, followed by viral warts and contact dermatitis, at 91.8% each. Conversely, only 29.5% correctly diagnosed cutaneous candidiasis, followed by basal cell carcinoma and seborrheic keratosis, at 31.1% and 32.8%, respectively (Table 4).

### 3.4. Management Plan

Along with the proportion of PCPs who had correct management, of 72.28%, the mean management score was 7.14 (±1.3). Most participants (93.4%) correctly managed psoriasis, followed by bacterial skin infections and acne vulgaris, at 93.4% and 91.8% of PCPs, respectively. However, melanocytic nevi were improperly managed by 75.4%. Additionally, only 32.8% and 37.7% correctly managed cutaneous candidiasis and melasma, respectively (Table 4). Moreover, participants older than 40 years and those interested in dermatology had significantly higher scores (*p* = 0.03 and *p* = 0.033, respectively) (Table 3).

### 3.5. Referral Decision

Among the 22 selected cases, only basal cell carcinoma and seborrheic keratosis were eligible to be referred, or when any other case was complicated, severe, or unresponsive to treatment as per the AAFP [9]. Consequently, the mean referral rate for 20 cases was 11.74 (±3.3). There were weak negative correlations between referral decisions and higher knowledge (r = −0.33, *p* = 0.01), correct diagnosis (r = −0.313, *p* = 0.014), and correct management (r = −0.296, *p* = 0.021).

Furthermore, the most commonly referred DC among the 22 cases was viral warts, with a referral rate of 96.7%, followed by a referral rate of 95.1% for basal cell carcinoma, seborrheic keratosis, and vitiligo. In contrast, contact dermatitis, pityriasis alba, and herpes simplex infection were the least frequently referred DCs, with referral rates of 3.3%, 23%, and 24.6%, respectively.

### 3.6. Encountering Proportion

The most commonly encountered DCs by PCPs were acne vulgaris, chicken pox, and Tenia Pedis, with encounter rates of 96.7%, 95.1%, and 91.8%, respectively. Compared to basal cell carcinoma, seborrheic keratosis and cutaneous leishmaniasis were the least encountered DCs, with encounter rates of 14.8%, 23%, and 31.1%, respectively.

## 4. Discussion

### 4.1. Summary

In this study, we assessed the knowledge of PCPs and their proficiencies in diagnosis, management, and referral decisions in the Jouf region regarding common DCs in Saudi Arabia. Additionally, this study investigated the barriers and facilitators in primary care settings to provide a holistic approach for patients with DCs. In the interviews, particular emphasis was placed on referrals, prescribed drugs, and the kind of medical advice patients should receive.

The pattern of DCs encountered by PCPs in this study was consistent with what PCPs usually face [1,2,3,4,5,6,7,15,16,17]. The mean knowledge level among PCPs in the Jouf region was 7.08 (±1.3). This level was higher than that in most of the literature. Possible explanations may include our small sample size, the difference in the participants’ characteristics in this study compared to previous studies, and the fact that our assessment tool was peculiar to our study [2,7,8,9,10,11,12,13,14,18,19,20,21,22,23].

For instance, several studies investigating PCPs’ knowledge and practice in dermatology have been carried out on various objectives, including eczema [12], topical corticosteroids [24], suspicious pigmented skin lesions [20,22], atopic dermatitis [25], and psoriasis [21]. At the same time, other studies evaluated the prevalence of DCs in PHC settings [1,4,7,15,16,17], PCPs’ knowledge [2,11,19,20], different approaches to management [18,21,22,23,24], and referral strategies [26,27,28,29,30,31,32].

### 4.2. Knowledge

We found that 83.6% of participants had acceptable to good knowledge. However, the proportion of correct answers on different parameters varied widely throughout the studied cases; such a result has not previously been reported. Previous studies concluded that PCPs scored poorly regarding common skin disorders [11,19]. This outcome may be explained by the fact that in our study, almost two-thirds of participants had formal postgraduate degrees and had more years of experience, the latter being statistically significant for higher knowledge.

Interviews with PCPs gave us insight into the resources used when managing dermatological conditions, including consultations, follow-up, clinical experience, and the Internet. “I usually use internet resources as they are up to date on the evidence-based approach” (PCP 2, male resident family physician, two years of experience).

Most of the DC groups that had higher overall knowledge (such as about disorders of skin appendages, dermatitis and eczema, and skin infections) were among the most prevalent categories in the country, as reported in Almohideb’s 2020 systematic review and meta-analysis [3].

This may suggest that PCPs with more exposure to common DCs would have better knowledge of these conditions. Moreover, exposure to DCs has been mentioned as an enhancer in PCP expertise. “The exposure and experience also play a major role. In our center, we see a lot of dermatological cases every day” (PCP 2, male resident family physician, two years of experience).

### 4.3. Disease Recognition

Our study’s mean diagnosis score was 7.01 (±1.51), which is higher than that in previous studies [27,33]. However, the proportion of correct diagnoses in each case is consistent with Moreno’s [33]. Although more than two-thirds of PCPs misdiagnosed basal cell carcinoma, almost all of them would refer it as guided by the AAFP [9]. This may suggest that PCPs would probably refer some conditions to reduce their diagnostic uncertainty when identifying red flags.

This finding is further supported by Rübsam et al., who found that PCPs would take various approaches to reduce diagnostic uncertainty by ruling out red flags, therapeutic trials, close follow-ups, and specialist consultation or referral [8].

The main issue of concern for PCPs was a lack of facilities and diagnostic tools needed to reach a diagnosis. “In most cases, we refer patients because we could not diagnose the case, which is due to lack of examination tools, strong similarities between different conditions, or unavailability of certain lab investigations” (PCP 2, male resident family physician, two years of experience). Hence, the availability of simple diagnostic tools in PHCs might decrease healthcare costs by lowering referrals to secondary providers [28,29].

Ramsay and Fox adopted a broader perspective in 1981, as they evaluated the capability of PCPs to accurately diagnose 20 frequently encountered DCs and compared their results with dermatologists taking the same examination [27]. However, PCPs could accurately recognize 54% of lesions, whereas dermatologists achieved a mean score of 96% [27].

### 4.4. Management Plan

Our finding of PCPs who had correct management, of 72.28%, was higher than Al-Zahrani et al.’s result of 61% [11]. However, the observed decrease in the management of melanocytic nevi (24.6%) could be attributed to the increased suspicion of neoplastic potential and inaccurate diagnosis of the lesion. This may point to poor differentiation skills between benign and malignant skin lesions among our study population. In Chen’s study, PCPs would probably refer patients even with a lower suspicion of melanoma. This leads to potentially unnecessary referrals and a burden on secondary care [22].

However, this finding does not support the finding of Baade’s study, as they concluded that Australian GPs appropriately diagnosed and managed different pigmented skin lesions [20]. This could be attributed to lower exposure to pigmented skin lesions in the region, as previous research indicated that the prevalence of new cases of skin cancers presented to clinicians would increase their ability to diagnose and manage such cases [34,35].

In our interviews, we explored how PCPs manage common DCs. We found a consensus amongst our interviewees that topical corticosteroids, emollients, antifungals, and antibiotics were commonly prescribed during practice. “Topical corticosteroids and fusidic acid are the most medications we prescribe; also we usually prescribe emollients because we see a lot of atopic dermatitis cases among children… all medications we prescribe are covered by the ministry of health” (PCP 2, male resident family physician, two years of experience).

From the dermatologists’ point of view, improper management of DCs by PCPs was very rare, and when encountered, no or mild complications occurred. This was attributed to the disease course of common DCs: “Very rare, especially that common skin diseases rarely develop complications due to bad management” (Dermatologist 2, male senior registrar, six years of experience), along with making the referral decision when not knowing the diagnosis or proper management: “Improper management of skin problems by PCPs is very rare, due to avoidance behavior and referral” (Dermatologist 4, male senior registrar, five years of experience).

Some of the difficulties facing PCPs in managing DCs include patient compliance with the medication and follow-up needed to manage the disease. “Some of the difficulties are appointment scheduling and patient compliance to follow-ups” (PCP 1, male senior registrar family physician, five years of experience).

### 4.5. Referral Decision

Another important parameter of our study was evaluation of the PCPs’ referral decisions. The pattern of referred cases was consistent with what PCPs were found to usually refer in a previous prospective study by Moreno et al. [33].

In our study, the mean referral rate of 11.74 (±3.3) must be cautiously interpreted. This implies the probability of PCPs when facing such conditions if they would refer them, meaning this referral rate is not only limited to PCPs’ knowledge of the presented conditions, but also to the specific PHC utilities. However, to minimize this ambiguity, we investigated the barriers and difficulties facing PCPs in managing DCs in primary care settings. We found that even when PCPs suspect the correct diagnosis and are able to manage such a case, in practice, they may face barriers such as regularity issues and unavailability of equipment and treatments, leading to more unnecessary referrals to dermatologists.

Referral decisions were made based on multiple reasons; a lack of diagnostic tools was previously mentioned as one of them. Other reasons include the patient’s preference to see a dermatologist. “I would refer a patient if his desire was a referral even if I can manage such a case in a PHC” (PCP 3, male specialist family physician, 18 years of experience).

An unclear diagnosis was also mentioned as a significant reason to refer patients. “Some cases are not clear to diagnose, so we need a second opinion or a consultation, so we refer them” (PCP 4, female GP, 16 years of experience). The availability of drugs and even the failure thereof were some of the other reasons highlighted for referral. “I would refer the patient when the prescribed drug did not give the expected results” and “if the drug of choice is not available at the PHC” (PCP 1, male senior registrar family physician, five years of experience).

Our interviews showed many reasons why PCPs may refer DCs; severe cases are one of them. “For mild cases that we can treat, we would treat them, but for severe cases that need specialist intervention, we directly refer them” (PCP 4, female GP, 16 years of experience).

Multiple reasons were identified as to why some patients would prefer to go directly to specialists. Some of these were seeking a second opinion and patient misconception that PCPs cannot handle a DC efficiently. “Patients should be aware that their family physician is well trained with a professional level of skills, knowledge, and abilities to manage common health problems including the skin” (Dermatologist 3, male specialist/lecturer, six years of experience).

Another reason is that patients with DCs bypass PHCs: “Some patients believe that secondary and tertiary hospitals are more prepared with medications and laboratory investigations than PHCs” (Dermatologist 2, male senior registrar, six years of experience). Nevertheless, such facilities are only needed for complex cases, while nearly all of the common DCs are straightforward and can be managed in a reasonably equipped PHC.

Existing studies recognize the critical role played by PCPs as gatekeepers. Meanwhile, approximately 50% of referrals made by PCPs could have been managed in PHC settings [25]. However, as we mentioned previously, the referral practice is complex, and many clinical and non-clinical factors play a role in it. Furthermore, Imison demonstrated that the referral decision may differ from one PCP to another when seeing the same condition, and attributed that to a lack of skills and supporting infrastructure to manage the patient appropriately [36].

Accordingly, evidence for improving referral quality includes educational activities, referral guidelines and clear referral criteria, consultant feedback, organizational interference, and involving patients in decision making [33,36]. A referral management strategy in a similar fashion is believed to be the most cost- and clinically effective [36].

Moreover, we found that PCPs with higher knowledge, correct diagnosis, and correct management would refer fewer cases than other PCPs. This, again, may reflect the emphasis on continuous education and specified training for the area of practice of PCPs’, who are the main referrers to secondary and tertiary care. An increasing referral rate contributes to an increase in the burden on secondary care, resulting in longer patient waiting lists.

Subsequently, patients with severe conditions are prone to experiencing poorer health outcomes and lower patient satisfaction. Additionally, increases in health care expenditure are affected by increasing referral rates [28,37]. “Adherence of primary care centers to referral regulations will improve the waiting lists on specialized clinics” (Dermatologist 3, male specialist/ lecturer, six years of experience).

On the other hand, dermatologist interviewees stated that most referred cases required specialist intervention. “I encounter a variety of referred cases from PHCs. However, approximately 70% of them need dermatologist intervention, other 30% should have been managed by PCPs” (Dermatologist 1, male consultant, 13 years of experience).

This finding suggests that if we trained our PCPs on the common DCs in the region and supported them with all the infrastructure they need, they might refer fewer cases, resulting in better sequelae. In accordance with the present results, previous research demonstrated that the average percentage of DCs referred by PCPs decreased significantly, from 31.9% to 23.5%, after completing a short course in dermatology [38].

### 4.6. Training in Dermatology

In our study, training in dermatology was not significantly correlated with higher scores in any parameter comparable to previous literature [11]. Conversely, PCPs who did not have had training in dermatology scored higher, with a chi-square value of 2.9. This rather contradictory, unexpected result may be due to inconsistent training, improper training for PCPs’ practice, or failure to appropriately target the common conditions in the region.

Furthermore, all our interviewees agreed that there was a need for dermatological courses for PCPs. “We can read and teach ourselves, but it is better when it comes from a dermatologist, especially on common dermatological conditions” (PCP 3, male specialist family physician, 18 years of experience). Additionally, the need for dermatology rotations in PHCs and vice versa was mentioned. “Dermatologists visits to PHCs or clinics at PHCs once every week or two weeks, PCPs rotations in dermatology clinics are needed” (PCP 1, male senior registrar family physician, five years of experience).

Nevertheless, several studies have underlined the growing demand for dermatology training for PCPs [15,19,20,21,22,23], where PCPs reported that lack of training is the primary obstacle to maintaining such a level of care [11,12,19]. On the other hand, other studies have emphasized the need for training of PCPs in dermatology to reinforce referral guidelines, which can significantly enhance the quality and appropriateness of referrals [30,39]. For instance, Foot et al. demonstrate the dimensions of high-quality referrals, which include the necessity, process quality, timeliness, destination, and content of referrals [32].

However, PCPs were shown to suffer from high workloads and consequently do not have time for self-improvement. “The high workload makes it difficult to have time to attend such courses and workshops” (PCP 4, female GP, 16 years of experience).

## 5. Strengths and Limitations

Various methods have been developed and established to understand the proficiency of PCPs in dermatology, as some authors analyzed data through self-administered questionnaires containing photograph-based MCQs regarding selected DCs [2,19,20,22]. Other researchers, however, have evaluated the 20 most common DCs and found more generalized results on PCPs’ knowledge and proficiency [27].

In accordance with the procedures carried out in the literature, we developed our study tool to evaluate all the domains of knowledge for all the selected cases. Hence, we took a mixed methodological approach to comprehensively understand the study findings. The nature of the chosen investigations reflects the real-life practice of PCPs in the Jouf region’s PHCs.

Nevertheless, the strength of our study, along with its mixed methodological nature, is the measures undertaken to ensure the validity and reliability of the study tool. However, our study has several limitations, which could influence the interpretation of the findings and their applicability to a broader context.

The small sample size was the major limitation. However, the needed sample of 103 participants could not be reached due to several barriers. Some PCPs were on vacation during data collection, covering school visits, on duty in the Ministry of Health Call Center, or allocated to the 24 h COVID-19 centers.

Furthermore, the use of convenience sampling, with almost two-thirds of participants having postgraduate degrees, could have led to selection bias. Additionally, there could be non-respondent bias where PCPs who are interested in dermatology possibly tended to complete the survey.

Other limitations were also met in the interview section. Only four PCPs were interviewed and were selected from the main local PHC. Hence, their responses may not necessarily reflect PCPs’ views in the region.

## 6. Implications and Future Research

We have deduced through our research that PCPs need more training covering all the common DCs, especially the ones they had deficits in, as presented by our findings. This can be accomplished by improving medical schools’ curricula and providing lectures and workshops on common local DCs by dermatologists. Additionally, PCPs need to recognize the referral guidelines to avoid unnecessary referrals.

Our finding of PCPs’ poor differentiation skills between benign and malignant skin lesions raises attention about the need for specialized training in such cases. Furthermore, PCPs need to be trained to perform screening examinations for such skin lesions to reach an early diagnosis and intervention, which in turn reduces the morbidity and mortality of such lesions.

We recommend the Ministry of Health provides the needed diagnostic tools and improves the PHC facilities. A lack of these capacities was the major contributing factor to unnecessary referrals that could otherwise be managed efficiently in the PHC setting.

We also recommend increasing public awareness about the role and expertise of PCPs in treating common DCs through campaigns and other educational measures. As mentioned earlier, it is not uncommon for patients to ask directly for a referral mainly due to patient misconceptions about their PCP.

An important step for future research on this issue would be to increase the sample size of PCPs. This would ensure a more representative deduction of PCPs’ approach to various DCs. Additionally, further studies are required on the facilities that PCPs need to appropriately manage DCs. We recommend that the number of PCPs interviewees should be increased and that they should come from more varied centers. This would help to gather more insight into the common issues that PCPs face.

## 7. Conclusions

We identified educational and regulatory aspects of PCPs’ clinical management of common DCs in the Jouf region. Our PCPs encounter a variety of DCs, and evidence was found to suggest that PCPs with more exposure and experience with common DCs would have better knowledge regarding these conditions and consequently refer fewer cases. Most PCPs demonstrated sufficient knowledge of common DCs. However, the referral practice is complex, and many clinical and non-clinical factors play a role in it. We identified multiple reasons for making a referral decision: lack of diagnostic tools, patient preference, diagnostic uncertainty, availability of drugs, failure of initial management, and severe cases. These aspects could be further optimized at the PCP, PHC, and patient levels to provide professional dermatology care.

## Figures and Tables

**Table 1:** Classification of the selected 22 skin related diagnoses according to the *ICD-10*.

**A. Dermatitis and eczema. L20–L30**	**iii. Protozoal Diseases: B50–B64**
Contact dermatitis. *L23–L25*	Cutaneous leishmaniasis. *B55.1*
Seborrheic dermatitis. *L21*	**iv. Infections of the skin and subcutaneous tissue: L00–L08**
Pityriasis alba. *L30.5*	Bacterial skin infections. *L01, L02, L03, A46*
**B. Disorders of skin appendages. L60–L75**	**D. Pigmentary disorders: L80–L99 and D22**
Alopecia Areata. *L63–L66*	Melanocytic nevi. *D22.9*
Acne Vulgaris. *L70*	Post inflammatory pigmentation. *L81.0 and L81.9*
**C. Skin infections:**	Vitiligo. *L80*
**i. Viral Skin Infections: B00–B9**	Melasma. *L81.1*
Herpetic infections. *B00*	**E. Papulosquamous disorders: L40–L45**
Chicken pox. *B01*	Psoriasis. *L40.9*
Viral warts. *B07*	Lichen planus. *L43.9*
**ii. Mycosis: B35–B49**	**F. Benign skin neoplasms: D23**
Tinea Pedis. *B35*	Seborrheic Keratosis. *D23*
Tinea Versicolor. *B35*	**G. Melanoma and other malignant neoplasms of skin: C43–C44**
Onychomycosis. *B35*	Basal cell carcinoma. *C44.91*
Cutaneous Candidiasis. *B37.2*	

**Table 2:** Demographics of participants and their practices.

Variable	N (%)
**Age (years), Mean (±SD)**	41 (±7)
<40	29 (47.5)
≥40	32 (52.5)
**Gender**	
Male	38 (62.3)
Female	23 (37.7)
**Nationality**	
Saudi	8 (13.1)
Non-Saudi	53 (86.9)
**Last Qualification**	
MBBS/MD	25 (41.0)
MRCGP	2 (3.3)
Diploma	9 (14.8)
Masters	4 (6.6)
Board Certified	15 (24.6)
Others	6 (9.8)
**Years in Practice, Mean (±SD)**	12 (±7)
<5 years	7 (11.5)
≥5 years	54 (88.5)
**Number of dermatological cases seen per month, Mean (±SD)**	27 (±25)
**Interested in Dermatology**	
Yes	36 (59.0)
No	25 (41.0)
**Had extracurricular training in dermatology**	
Yes	26 (42.6)
No	35 (57.4)

**Table 3:** Knowledge, diagnosis, and management scores among the study participants.

Total *N* ^a^ = 61 Variable (n)	Total *N* = 46		Total *N* = 49		Total *N =* 51	
	Diagnosis, n (%)	*p*-Value *	Management, n (%)	*p*-Value *	Overall Knowledge, n (%)	*p*-Value *
Age		0.874		0.030		0.488
<40 years (29)	22 (36.1)		20 (32.8)		23 (37.7)	
≥40 years (32)	24 (39.3)		29 (47.5)		28 (45.9)	
Gender		0.341		0.556		0.802
Male (38)	28 (45.9)		32 (52.5)		32 (52.5)	
Female (23)	18 (29.5)		17 (27.9)		19 (31.1)	
Qualification		0.530		0.155		0.568
MBBS/MD (25)	17 (27.9)		19 (31.1)		20 (32.8)	
Post-Graduate (36)	29 (47.5)		30 (49.2)		31 (50.8)	
Years in Practice, Mean (±SD)		0.325		**0.032**		**0.042**
<5 years (7)	6 (9.8)		6 (9.8)		6 (9.8)	
≥5 years (54)	40 (65.6)		43 (70.5)		45 (73.8)	
Interested in Dermatology		0.054		**0.033**		0.093
Yes (36)	27 (44.3)		32 (52.5)		32 (52.5)	
No (25)	19 (31.1)		17 (27.9)		19 (31.1)	
Had training in Dermatology		0.143		0.233		0.062
Yes (26)	17 (27.9)		19 (31.1)		20 (32.8)	
No (35)	29 (47.5)		30 (49.2)		31 (50.8)	

The above data represent the number (%) of participants who scored good–acceptable scores in each category. ^a^ The whole study population. * *p*-values of 0.05 or less are considered statistically significant and shown in bold text.

**Table 4:** The evaluated 22 cases and proportions of participants’ responses to the study parameters.

Skin Related Diagnosis and *ICD-10 Codes*		Proportion of Correct Diagnoses	Proportion of Correct Management	Referral Decision Taken	Encountering Rate	Knowledge Level
1	Acne Vulgaris. *L70*	98.4%	91.8%	52.5%	96.7%	95.1%
2	Chicken pox. *B01*	88.5%	91.8%	14.8%	95.1%	90.15%
3	Tinea Pedis. *B35*	86.9%	90.2%	16.4%	91.8%	88.55%
4	Viral warts. *B07*	91.8%	85.2%	96.7%	78.7%	88.5%
5	Contact dermatitis. *L23–L25*	91.8%	82.0%	3.3%	90.2%	86.9%
6	Psoriasis. *L40.9*	80.3%	93.4%	90.2%	77%	86.85%
7	Onychomycosis. *B35*	86.9%	85.2%	59%	68.9%	86.05%
8	Bacterial skin infections. *L01, L02, L03, A46*	77.0%	93.4%	59%	63.9%	85.2%
9	Tinea Versicolor. *B35*	77%	83.6%	41%	68.9%	80.3%
10	Alopecia Areata. *L63–L66*	85.2%	73.8%	82.0%	75.4%	79.5%
11	Pityriasis alba. *L30.5*	80.3%	65.6%	23.0%	86.9%	72.95%
12	Post-inflammatory pigmentation. *L81.0 and L81.9*	73.8%	70.5%	55.7%	45.9%	72.15%
13	Seborrheic dermatitis. *L21*	60.7%	82.0%	54.1%	72.1%	71.35%
14	Lichen planus. *L43.9*	68.9%	68.9%	90.2%	32.8%	68.9%
15	Vitiligo. *L80*	82.0%	55.7%	95.1%	68.9%	68.85%
16	Melasma. *L81.1*	82.0%	37.7%	82.0%	59%	59.85%
17	Seborrheic Keratosis. D23	32.8%	85.2%	95.1%	23%	59%
18	Basal cell carcinoma. *C44.91*	31.1%	83.6%	95.1%	14.8%	57.35%
19	Cutaneous leishmaniasis. *B55.1*	57.4%	54.1%	78.7%	31.1%	55.75%
20	Herpetic infections. *B00*	45.9%	39.3%	24.6%	82%	42.6%
21	Cutaneous Candidiasis. *B37.2*	29.5%	32.8%	62.3%	34.4%	31.15%
22	Melanocytic nevi. *D22.9*	34.4%	24.6%	93.4%	37.7%	29.5%
	Total	70.11%	71.38%	62%	63.41%	70.75%

## Data Availability

Data are available on request from the corresponding author.

## Abbreviations

DCs	Dermatological Conditions
PCPs	Primary Care Physicians
PHCs	Primary Healthcare Centers
AAFP	American Academy of Family Physicians
MOH	Ministry of Health
